# Supplementary material for: Minimally Invasive Facetectomy and Fusion for Resection of Extensive Dumbbell Tumors in the Lumbar Spine
Source: Medicina (Kaunas). 2022 Nov 8;58(11):1613. doi: 10.3390/medicina58111613 (PMC9694687; doi:10.3390/medicina58111613)
Supplement: Supplementary file 1 [file medicina-58-01613-s001.zip › medicina-1994630-supplementary.pdf]

Supplement Table S1: patients' data, LOS: length of hospital stay, EOR: extents of resection, GTR: gross total resection, STR: subtotal resection.

patient	surgery	Age (years)	Pathology	Duration of surgery (h)	Eden Type	Tumor Volume (ml)	Treatment status	Blood loss (ml)	EOR	Location	Macnab score	LOS (days)	Symptoms
1	Facet-sparing	43	Schwannoma (WHO grade I)	2,3	3	28,9	Progression	50	STR	L 2-3	4	4	Back pain, sciatica
2	Facetectomy	62	Hemangiopericytoma (WHO grade II)	4,2	3	9,64	Progression	1600	GTR	D 12-L 1	4	6	incomplete brown sequard
3	Facet-sparing	62	Hemangiopericytoma (WHO grade II)	4,2	3	39,1	De novo	1500	STR	D 12-L 1	3	8	Back pain, sciatica
4	Facetectomy	29	Schwannoma (WHO grade I)	3,7	3	2,16	De novo	50	GTR	L 3-4	4	3	Radiculopathy
5	Facetectomy	56	Schwannoma (WHO grade I)	5,2	3	19,40	De novo	1100	GTR	L 3-4	4	9	Back pain, radiculopathy
6	facetectomy	50	Schwannoma (WHO grade I)	5,1	3	23,8	Progression	150	GTR	L 2-3	4	5	Radiculopathy
7	Facet-sparing	38	Schwannoma (WHO grade I)	0,6	3	48,5	De novo	200	STR	L 2-3	5	6	Back pain, sciatica
8	Facetectomy	41	Schwannoma (WHO grade I)	5,2	3	64,9	De novo	900	GTR	D 11-L 1	5	*5*	Radiculopathy
9	Facetectomy	38	Schwannoma (WHO grade 1)	3,3	3	3,35	Progression	50	GTR	L 3-4	4	7	Radiculopathy
10	Facetectomy	62	Schwannoma (WHO grade I)	5,4	2	2,70	Progression	800	GTR	L 3-4	4	*5*	Radiculopathy
11	Facet-sparing	55	Schwannoma (WHO grade I)	5,0	2	4,37	De novo	50	STR	L 3-4	4	8	Back pain, sciatica
12	Facet-sparing	31	Schwannoma (WHO grade I)	3,8	3	7,45	De novo	300	GTR	L 1-2	5	8	Backpain, sciatica
13	Facet-sparing	33	Schwannoma (WHO grade I)	1,7	3	2,65	De novo	100	STR	L 2.3	4	5	Back pain, sciatica

14	Facet-sparing	34	Schwannoma (WHO grade I)	3,6	3	1,58	Progression	50	STR	L 2-3	2	14	Back pain, sciatica
15	Facetectomy	49	Schwannoma (WHO grade I)	3,6	2	8,09	Progression	200	GTR	L 2-3	5	2	Back pain
16	Facet-sparing	46	Schwannoma (WHO grade I)	2,5	2	17,1	De novo	50	STR	L 2-3	4	4	Back pain, sciatica
17	Facetectomy	23	Schwannoma (WHO grade I)	2,3	3	1,96	Progression	200	GTR	D 12- L 1	5	3	Back pain
18	Facet-sparing	21	Schwannoma (WHO grade I)	6,3	3	7,75	De novo	800	STR	D 12- L 1	5	6	Back pain, sciatica
19	Facetectomy	31	Schwannoma (WHO grade I)	4,1	3	11,40	De novo	50	GTR	L 2-3	5	4	Back pain
